# Supplementary material for: Diagnosis and Early Prediction of Lymphoma Using High-Throughput Clonality Analysis of Bovine Leukemia Virus-Infected Cells
Source: Microbiol Spectr. 2022 Oct 13;10(6):e02595-22. doi: 10.1128/spectrum.02595-22 (PMC9769566; doi:10.1128/spectrum.02595-22)
Supplement: Supplemental file 1 — Fig. S1 to S3. Download spectrum.02595-22-s0001.pdf, PDF file, 0.2 MB [file spectrum.02595-22-s0001.pdf]

Supplemental Figure 1

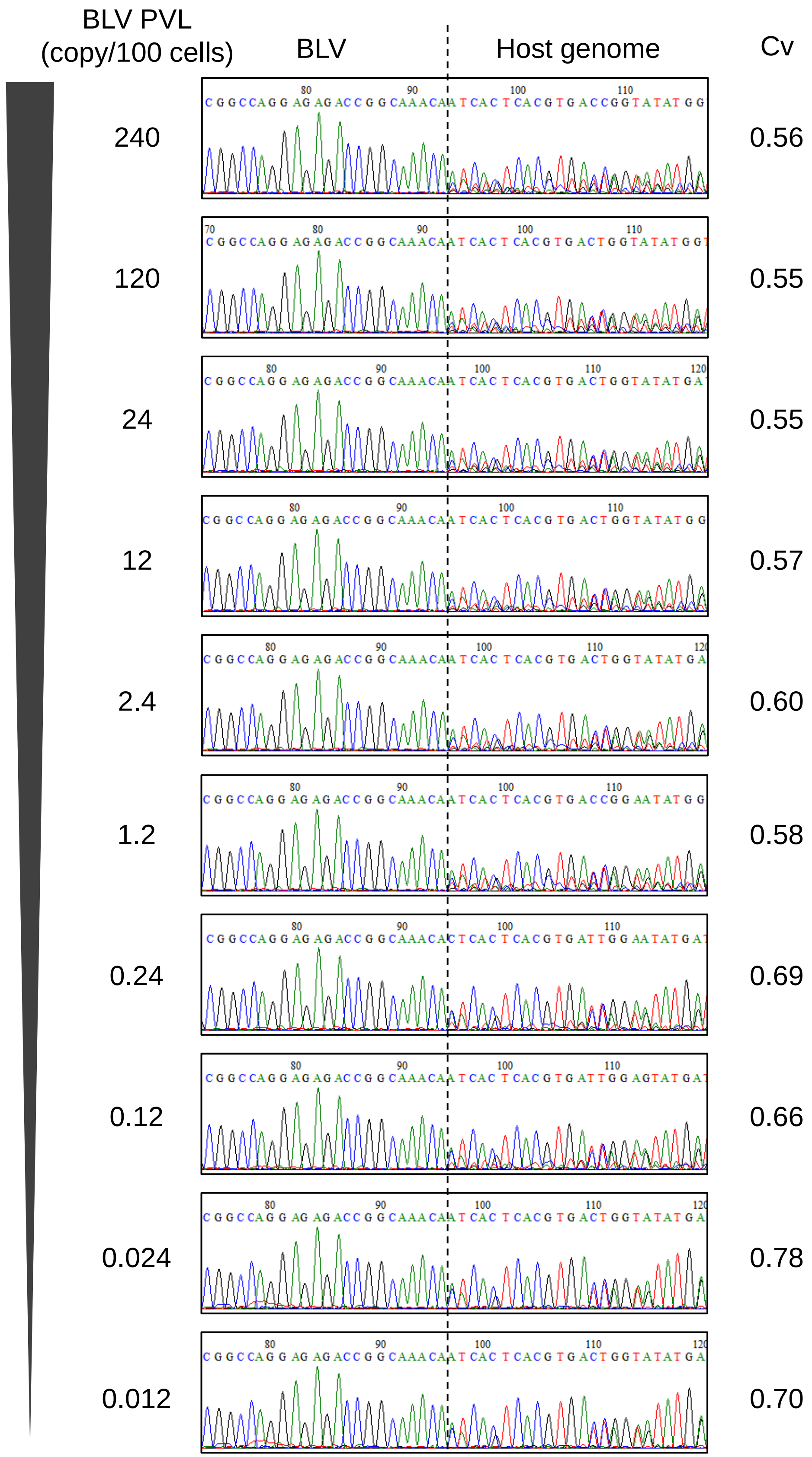

Supplemental Fig. 1. Sanger sequence spectrum of a dilution series of BLV-infected cell line by RAISING.

Sangar sequence spectrum of amplicons of RAISING using a dilution series of BLV-infected cell line (BL3.1) were shown with BLV PVL measured by qPCR and Cv analyzed by CLOVA. Dashed line indicates the position of the BLV integration site.

Supplemental Figure 2

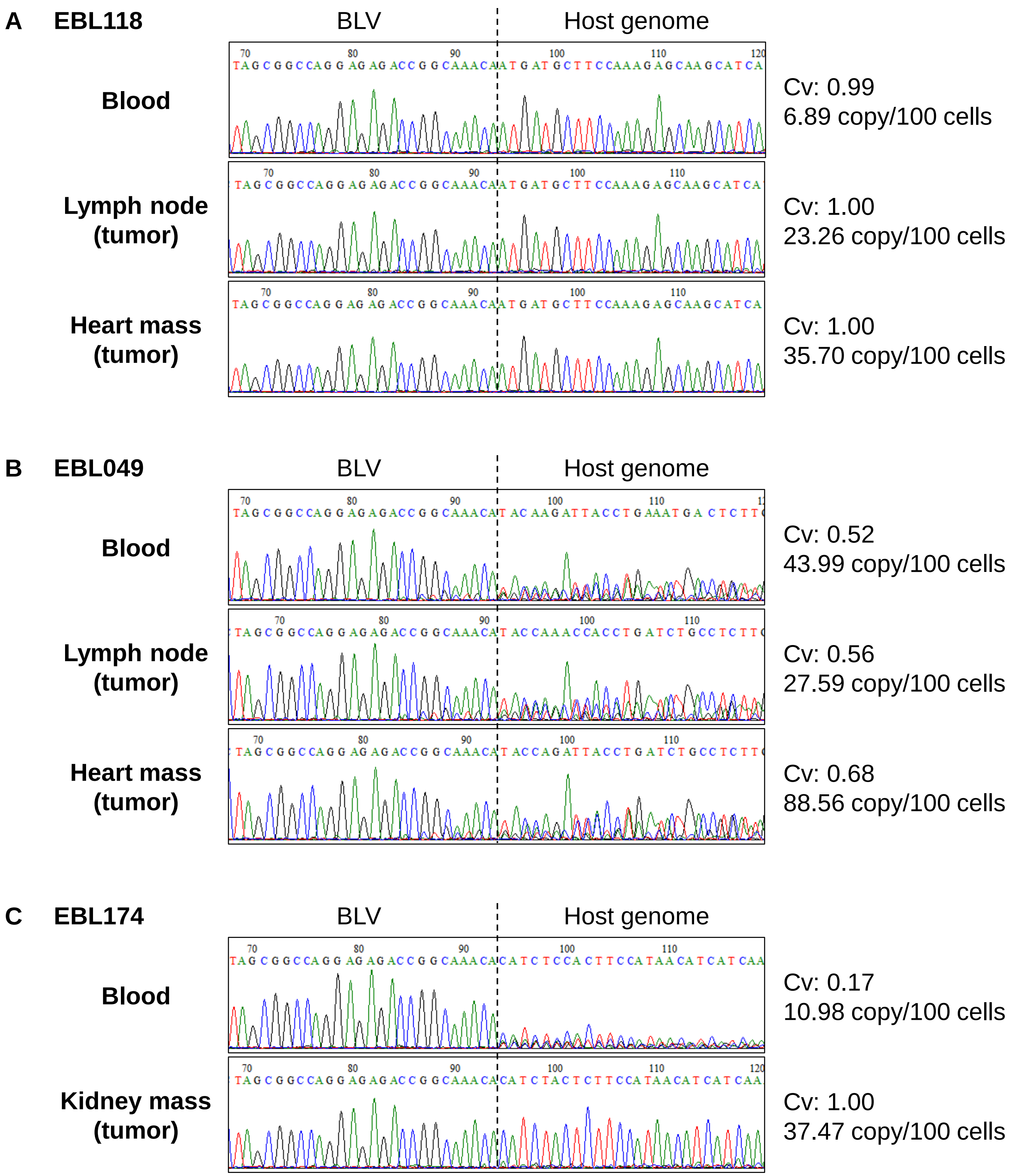

Supplemental Fig. 2. Comparison of integration sites analyzed by RAISING-CLOVA in tumor and blood samples of EBL cattle.

(A─C) Sangar sequence spectrum of representative blood and tumor samples from EBL cattle (A, EBL118; B, EBL049; C, EBL174) were shown with Cv and BLV PVL. Dashed line indicates the position of the BLV integration site.

Supplemental Figure 3

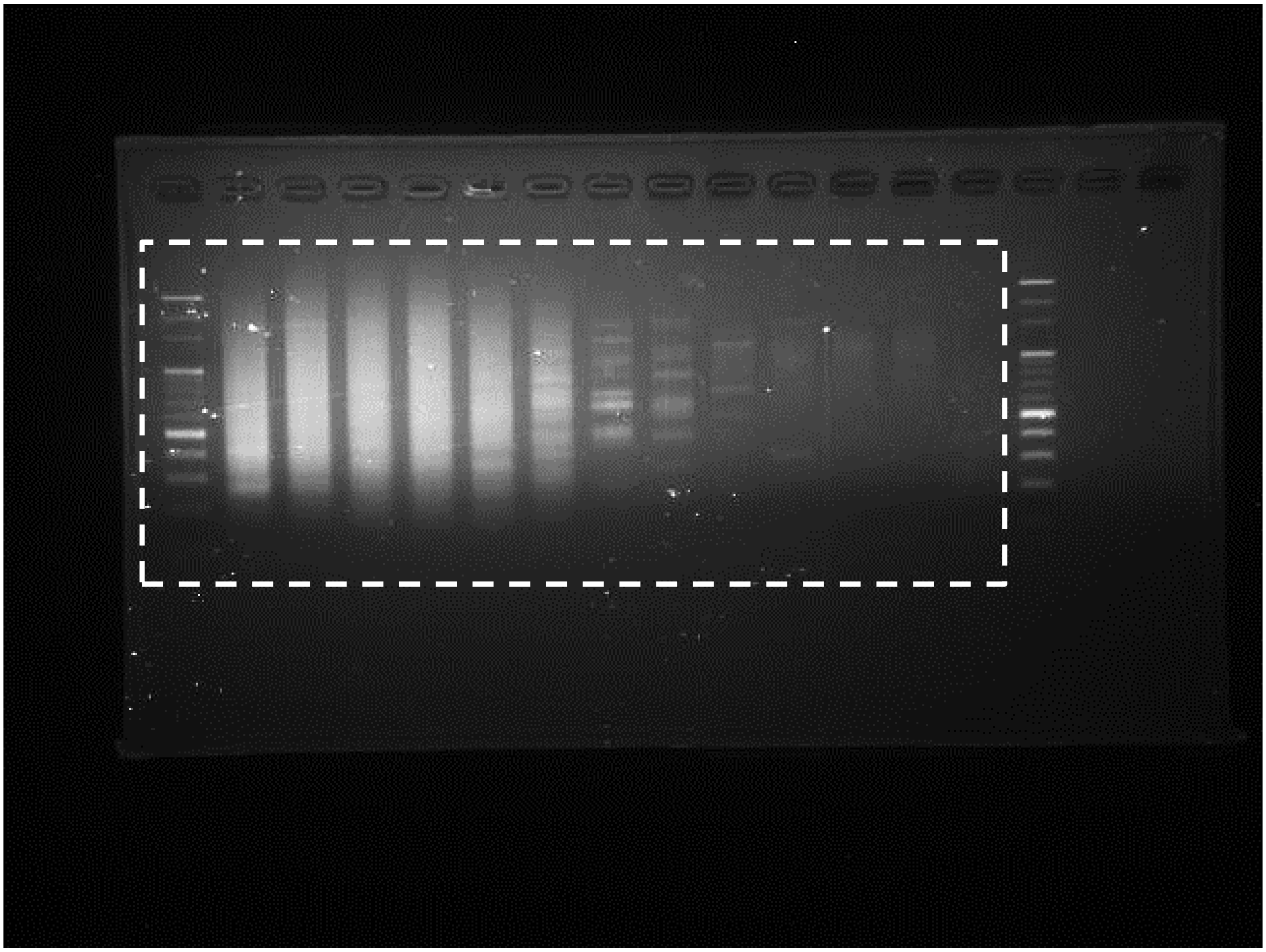

Supplemental Fig. 3. An uncropped gel image for Fig. 1a.
